# Effects of Oral Multi-Vitamin Multi-Mineral Supplement Formulations on Laboratory Outcomes and Quality of Life: A Quasi-Experimental Study

**DOI:** 10.3389/fnut.2022.889910

**Published:** 2022-06-27

**Authors:** Nawin Jittat, Krit Pongpirul, Bhakanij Tepwituksakit, Pratchayada Iammaleerat, Julia Heath, Palita Lungchukiet, Nimit Taechakraichana, Artirat Charukitpipat

**Affiliations:** ^1^Bumrungrad International Hospital, Bangkok, Thailand; ^2^Faculty of Medicine, Chulalongkorn University, Bangkok, Thailand; ^3^Johns Hopkins Bloomberg School of Public Health, Baltimore, MD, United States; ^4^Asia Global Research Co., Ltd., Bangkok, Thailand

**Keywords:** multi-vitamin supplement, quality of life, drug formulations, quasi-experiment design, multi-mineral supplement

## Abstract

**Background:**

Multi-vitamin multi-mineral (MVMM) products often come in several single-substance capsules from different manufacturers. However, attempts to mix several vitamins and minerals into one MVMM product have been complicated and often involve legal concerns. This study aimed to comparatively investigate the changes in laboratory parameters and the quality of life (QOL) among individuals who received different MVMM formulations.

**Methods:**

This three-arm non-randomized controlled trial was conducted at VitalLife Scientific Wellness Center (VSWC), Bangkok, Thailand. A total of 72 healthy adult individuals with total serum 25-(OH)D level of 20–29 ng/ml were invited to choose from the three available options, namely, (1) Hydro-Cell-Key (HCK®, Hepart AG, Switzerland) contains vitamin D3 2,000 IU, vitamin C 1,000 mg, vitamin E 166 mg, vitamin A 1 mg, coenzyme Q10 30 mg, natural carotenoids 8 mg, and citrus flavonoids 200 mg in granule formulation; (2) VTL-7 (VWSC) contains similar vitamins and minerals but in capsule formulation; and (3) placebo capsule (no supplement). The 36-Item Short-Form Health Survey (SF-36) was used to measure QOL at baseline, month 3 and 6. A generalized estimating equation (GEE) was used to compare the repeated-measure outcomes across the three groups. This study was registered at the Thai Clinical Trial Registration (TCTR20190205002) and approved by the Bumrungrad International Institutional Review Board (BI-IRB No.258-10-18PhFub).

**Results:**

Both VTL-7 and HCK saw a significantly higher increase in vitamin D than placebo at months 3 and 6, i.e., VTL-7 from 25.15 ± 2.13 to 35.53 ± 6.11 (*p* < 0.001) and 33.38 ± 6.89 (*p* < 0.001); HCK from 24.25 ± 3.08 to 28.43 ± 5.93 (*p* = 0.005) and 27.40 ± 5.24 (*p* = 0.012); and placebo from 24.00 ± 2.73 to 23.05 ± 4.39 (*p* = 0.273) and 22.30 ± 6.23 (*p* = 0.200), respectively. Similarly, β-carotenoids of VTL-7 vs. HCK groups significantly increased from 0.88 ± 0.68 vs. 0.94 ± 0.55 at baseline to 3.03 ± 1.79 (*p* < 0.001) vs. 1.09 ± 0.61 (*p* = 0.125) and 3.26 ± 1.74 (*p* < 0.001) vs. 1.15 ± 0.66 (*p* = 0.064), respectively. These findings were corroborated through the GEE analysis. Other micronutrients at months 3 and 6 did not increase significantly from baseline in any group. The overall QOL among the three groups in terms of physical (*p* = 0.560) and mental (*p* = 0.750) health increased but was not statistically significant.

**Conclusion:**

The supplements of MVMM in capsule formulation increased the serum levels of some micronutrients to a higher extent than that of granule formulation. Participant adherence remains a potential confounder and should be further explored.

**Clinical Trial Registration:**

identifier: TCTR20190205002.

## Background

The human diet requires both macronutrients, including carbohydrates, proteins, and fats, and micronutrients, such as vitamins and minerals. While macronutrients provide the main source of calories, micronutrients are required for developmental processes. Even though they are only required in a small amount, they have a profound impact on health and are vital to body development, disease prevention, immune function, tissue regeneration, and optimizing health (1, 2).

Micronutrient supplementation has gained popularity among individuals who want to ensure and maintain their health and wellness. Consumed by 50% of adults and one-third of children in economically advanced economies, the dietary supplement business is worth more than US$100 billion annually (3). According to the National Health and Nutrition Examination Survey (NHANES) collected between 1999 and 2014, the prevalence of any supplement use has significantly increased from 52 to 58%, whereas that of vitamin and mineral use has increased from 47 to 52% and from 47 to 51%, respectively (4). The trend of any supplement use varied by age, sex, race/ethnicity, or education but not by diabetes duration or comorbidities associated with diabetes. During 2017–2018, 57.6% of adults of at least 20 years of age reported that they had taken a dietary supplement within the past 30 days; individuals with a higher family income were more likely to consume a dietary supplement than those with a lower family income (4). Furthermore, women were significantly more likely than men to use a dietary supplement overall (63.8 vs. 50.8%) (4).

In contrast to mono-substance supplements, supplementation of multiple vitamins and minerals has significantly decreased from 36 to 32%, largely because micronutrient supplements are often manufactured in numerous single-substance capsules, which can decrease compliance. Multi-vitamin multi-mineral (MVMM) supplements, which contain numerous vitamins and minerals within one single-substance capsule, alleviate the issue of non-compliance by making it easier for consumers to supplement with multiple vitamins and minerals. However, attempts to mix several vitamins and minerals into one MVMM product have been both legally and technically complicated. Each MVMM “recipe” must be registered with the national authority of the country, such as the Food and Drug Administration (FDA) (5). Furthermore, product variation of available MVMM supplements, including varying product formulations, has led to limited evidence on the efficacy of different forms of MVMM supplements. It has been suggested that different MVMM formulations could also impact the degree of nutrient absorption in the body. There is some evidence to suggest that vitamin D buccal spray, for instance, has higher absorption than soft gel capsules (6). However, the lack of evidence looking at the formulation of MVMM supplementation and its effects on laboratory parameters makes it difficult to understand the extent to which MVMM formulation impacts its efficacy. Thus, the objective of this study is to comparatively investigate the effectiveness of different formulations of combined MVMM products, specifically Hydro-Cell-Key (HCK) granules vs. VTL-7 capsules, and their impacts on certain laboratory parameters, including serum micronutrient levels and quality of life (QOL) (focusing on the vitality domain).

A hospital with a qualified production facility might procure raw substances and produce an MVMM product that does not require FDA approval for in-house use. With this in mind, Vital-Life Scientific Wellness Center (VSWC), i.e., a medical anti-aging and wellness center in Bangkok, Thailand, developed an in-house personalized vital-life (VTL-7) MVMM capsules. As several patients preferred fewer capsules per day, along with an absorption concern, VSWC considered switching from capsule to granule formulation. To compare the efficacy of a similar MVMM product in granule formulation, the HCK®, i.e., MVMM granules of the Hepart AG Switzerland group, was included in the study. The HCK granule contains vitamin D3, vitamin C, vitamin E, vitamin A, coenzyme Q10, carotenoids, and flavonoids; hence, it was determined to be one of the best available candidates in comparison with the VTL-7 capsules. Built into a plant hydrocolloid matrix, the vitamins and minerals in granules are released in the intestines through the colloid film formed by the colloidal system after ingestion. This process mimics the absorption of micronutrients from natural fruits and vegetables, resulting in optimal nutrient distribution throughout the body, along with prolonged absorption over several hours, prevention of disturbances, and antagonism of various micronutrients. However, the formation of a dry granulation requires many more steps than capsule formulation.

The clinical efficacy (i.e., serum level of the micronutrients) of the original HCK® granules has been anticipated to be higher than conventional capsule formulation; however, there has been no comparative evidence of the changes in blood vitamins and minerals between the two products. As previously mentioned, product formulation could affect not only individual compliance but also laboratory changes. Thus, the methods used to examine both the effects of MVMM supplementation and the relationship between MVMM product formulation on laboratory parameters and QOL are presented in this study.

## Methods

### Study Design and Setting

This three-arm non-randomized controlled trial was conducted at VSWC, a medical anti-aging and wellness center, focusing on promoting good health and preventing illness and chronic diseases. It is a subsidiary company of Bumrungrad International Hospital Public Company Limited, i.e., one of the largest international hospitals for medical tourism located in Bangkok, Thailand.

### Participants

A total of 72 healthy adult individuals with insufficient levels of total serum 25-(OH)D level (20–29 ng/ml) were invited to participate in this study. Individuals with poorly controlled blood pressure, impaired kidney function (eGFR < 30 ml/min/1.73 m^2^), pregnancy, malabsorption, bowel surgery, and currently on medications or supplements that might affect the levels of vitamins were excluded from this study. Individuals who have underlying diseases that require vitamin D treatment (i.e., osteoporosis and hyperparathyroidism) or those who have a deficient level of total serum 25-(OH)D level (<20 ng/ml) were also excluded. As carotenoids could increase the risk of lung cancer (7), current and former smokers were also excluded.

### Supplements

**Figure 1:**
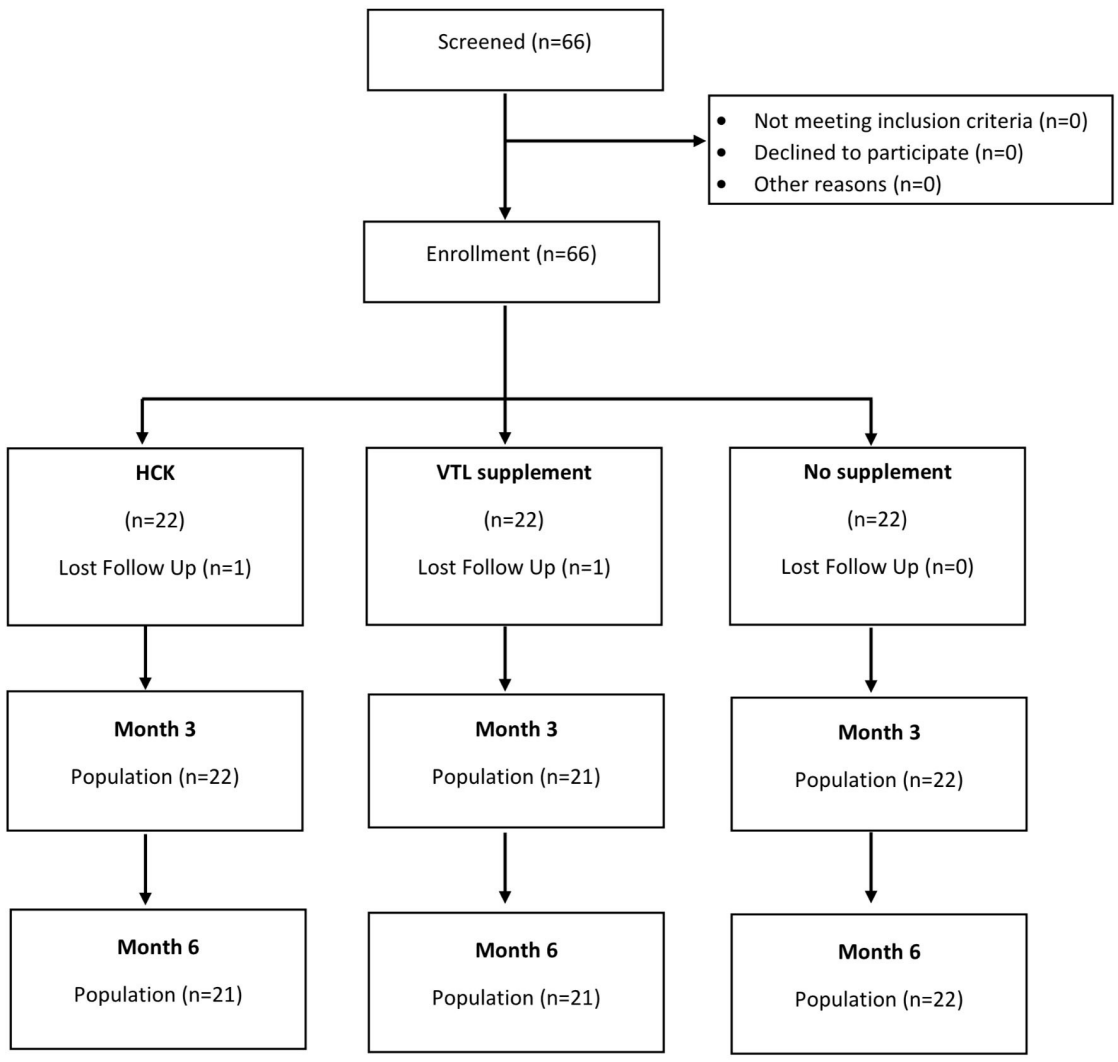
Patient disposition.

Participants were assigned to one of the three groups (Figure 1), namely, HCK® (Hepart AG, Switzerland), VTL-7 (VSWC, Thailand), and placebo group (no supplement). The two study groups have similar micronutrient compositions, namely, vitamin D3 2,000 IU, vitamin C 1,000 mg, vitamin E 166 mg, vitamin A 1 mg, coenzyme Q10 30 mg, natural carotenoids 8 mg, and citrus flavonoids 200 mg; HCK was prepared in granules, while VTL-7 was the capsule formulation of the HCK product. The placebo group was used to allow for a comparison of the effects of each MVMM formulation on specified lab parameters and QOL vs. no supplementation, in addition to providing comparative evidence between the two products. Diet counseling was not included for any group as a part of this trial.

### Outcomes

Primary outcomes (blood levels of the six micronutrients) and secondary outcomes (hs-CRP, homocysteine, lipid profile, ESR, CD4, CD8, and QOL) were measured at baseline, month 3, and 6. Although previous studies have found micronutrient supplementation to have a positive impact on overall mood and QOL, the 36-Items Short Form Health Survey (SF-36) was used to determine if different MVMM formulations impact the QOL in different ways (10–14). Circulatory cholesterol, lipoproteins, and homocysteine were also included as secondary outcomes since MVMM supplementation has been previously found to impact cholesterol levels and decrease homocysteine levels in certain populations (12, 18).

### Sample Size

Previous trials suggest that a weekly supplementation of 50,000 IU cholecalciferol for 12 weeks resulted in an increase of 25-(OH)D (8). Given the differences in 25-(OH)D levels between the intervention and placebo control groups of 7.90 ng/ml and a pooled standard deviation of 7.85 ng/ml, an alpha SD of 0.05%, and a beta SD of 0.20, approximately 16 subjects were required per arm. Assuming a conservative dropout rate of 30%, 22 subjects were anticipated for each arm of this study.

### Statistical Methods

Descriptive statistics (mean, standard deviation, and percentage) were used for demographic variables. The analysis of variance (ANOVA) and generalized estimating equation (GEE) were used to compare the changes in cross-sectional and longitudinal laboratory outcomes between the three groups, respectively. No subgroup analysis was performed. The data were analyzed based mainly on the intention to treat (ITT) principle, whereas per-protocol (PP) analysis was also performed to ensure the robustness of the analysis. In case of discrepant findings from both approaches, the ITT analysis was chosen.

### Trial Registration and Ethical Approval

This study was registered at the Thai Clinical Trial Registration (Registration No. TCTR20190205002) and approved by the Bumrungrad International Institutional Review Board (BI-IRB No. 258-10-18PhFub). All participants provided written informed consent.

## Results

### Characteristics of the Participants

**Figure 2:**
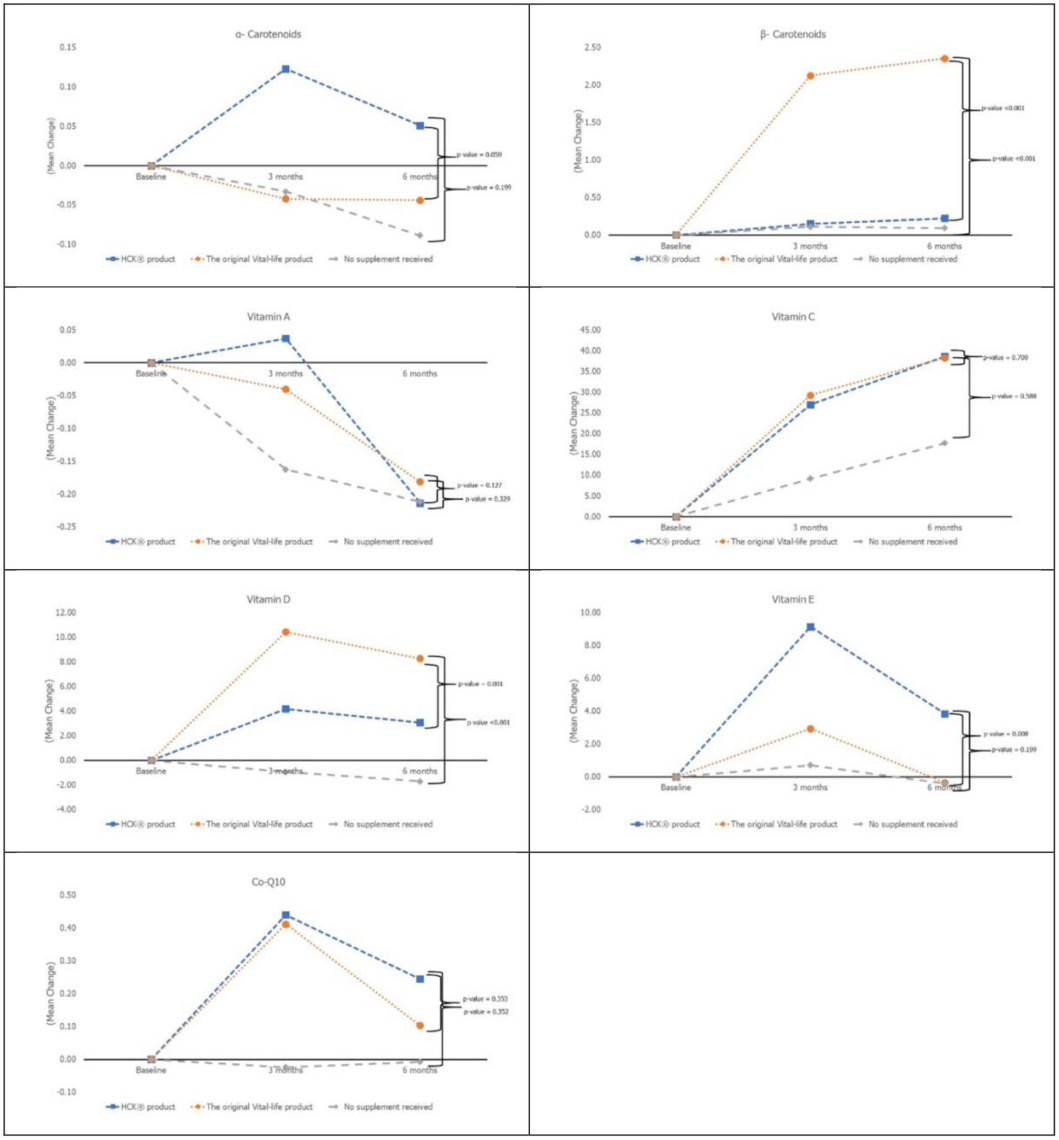
Micronutrient levels at baseline, month 3 and 6 among all 3 groups.

The age of the participants was 35.06 ± 8.47 years, 83.3% were women, and the BMI of the participants was 22.51 ± 3.57 (Table 1). While almost all the characteristics of the participants were not significantly different across the three groups, the placebo control group was significantly younger than the intervention groups (*p* = 0.037). The baseline micronutrient and biomarker levels were comparable across the three groups (Table 2 and Figures 1, 2).

**Table 1:** Characteristics of the participants.

	**Overall**	**HCK**	**VTL-7**	**Placebo**	***p*-value**
Age (years)	35.06 ± 8.47	37.64 ± 10.04	36.14 ± 7.09	31.41 ± 6.99	0.037
Women	83.30%	77.30%	77.30%	95.50%	0.175
Systolic BP (mm Hg)	109.14 ± 11.24	109.95 ± 11.39	111.64 ± 12.34	105.82 ± 9.51	0.212
Diastolic BP (mm Hg)	70.62 ± 8.72	71.27 ± 9.38	71.05 ± 9.73	69.55 ± 7.15	0.780
Pulse rate	76.45 ± 14.28	71.77 ± 10.55	76.50 ± 11.12	81.09 ± 18.73	0.095
Body temperature (°C)	36.75 ± 0.28	36.69 ± 0.18	36.90 ± 0.35	36.67 ± 0.24	0.120
Body weight (kg)	58.27 ± 11.26	58.12 ± 10.90	58.08 ± 13.16	58.60 ± 10.03	0.986
BMI	22.51 ± 3.57	21.95 ± 3.39	22.54 ± 3.75	23.05 ± 3.62	0.601

**Table 2:** Laboratory outcomes and quality of life at baseline, month 3 and 6.

	**Overall**	**HCK**	**VTL-7**	**Placebo**	*p*-value*
**Primary outcomes**					
**Vitamin D (ng/mL)**					
Month 0	24.47, 2.68	24.25, 3.08	25.15, 2.13	24.00, 2.73	0.325
Month 3	28.90, 7.47	28.43, 5.93	35.53, 6.11	23.05, 4.39	**<0.001**
Month 6	27.61, 7.60	27.40, 5.24	33.38, 6.89	22.30, 6.23	**<0.001**
**Vitamin C (μmol/L)**					
Month 0	88.99, 32.52	86.20, 28.35	87.71, 35.48	93.07, 34.40	0.768
Month 3	110.96, 24.62	113.22, 25.84	117.71, 21.93	102.25, 24.31	0.103
Month 6	120.24, 35.82	123.62, 37.45	126.74, 36.36	110.82, 33.29	0.306
**Vitamin E (mg/L)**					
Month 0	30.33, 7.86	32.45, 10.76	29.11, 5.81	29.42, 5.86	0.300
Month 3	34.58, 12.39	41.60, 16.35	31.89, 7.85	30.13, 7.89	**0.003**
Month 6	36.07, 9.50	28.60, 5.35	29.02, 7.90	31.19, 8.39	**0.004**
**Vitamin A (μmol/L)**					
Month 0	1.90, 0.53	2.05, 0.67	1.82, 0.52	1.83, 0.35	0.283
Month 3	1.86, 0.53	2.08, 0.64	1.82, 0.49	1.66, 0.33	**0.026**
Month 6	1.71, 0.46	1.85, 0.52	1.68, 0.48	1.62, 0.34	0.230
**α-Carotenoid (μmol/L)**					
Month 0	0.25, 0.20	0.27, 0.15	0.24, 0.24	0.25, 0.20	0.895
Month 3	0.27, 0.24	0.39, 0.32	0.20, 0.17	0.21, 0.13	**0.011**
Month 6	0.22, 0.20	0.31, 0.25	0.20, 0.19	0.16, 0.10	**0.024**
**β-Carotenoid (μmol/L)**					
Month 0	0.89, 0.55	0.94, 0.55	0.88, 0.68	0.84, 0.43	0.823
Month 3	1.67, 1.44	1.09, 0.61	3.03, 1.79	0.95, 0.44	**<0.001**
Month 6	1.76, 1.52	1.15, 0.66	3.26, 1.74	0.93, 0.52	**<0.001**
**Co-Q10 (μmol/L)**					
Month 0	1.61, 0.75	1.71, 0.89	1.55, 0.85	1.57, 0.48	0.765
Month 3	1.89, 0.96	2.15, 1.21	1.98, 0.94	1.55, 0.59	0.107
Month 6	1.73, 0.71	1.96, 0.72	1.67, 0.65	1.57, 0.74	0.181
**Secondary outcomes**					
**hs-CRP (mg/L)**					
Month 0	0.28, 0.60	0.25, 0.79	0.20, 0.33	0.40, 0.60	0.522
Month 3	0.19, 0.30	0.15, 0.15	0.16, 0.28	0.24, 0.43	0.559
Month 6	0.22, 0.37	0.18, 0.23	0.23, 0.39	0.25, 0.46	0.848
**Homocysteine (μmol/L)**					
Month 0	8.86, 2.27	9.01, 2.20	8.99, 2.39	8.60, 2.29	0.800
Month 3	8.67, 2.20	8.34, 2.13	9.15, 2.10	8.53, 2.38	0.466
Month 6	8.43, 2.02	8.09, 1.65	9.00, 2.31	8.20, 2.01	0.285
**Total cholesterol (mg/dL)**					
Month 0	202.22, 31.16	213.09, 34.34	190.44, 28.90	203.13, 26.85	0.052
Month 3	204.08, 28.68	201.38, 26.71	200.41, 27.55	210.30, 31.76	0.463
Month 6	200.54, 33.92	193.93, 31.14	194.59, 26.72	212.52, 40.22	0.123
**Triglyceride (mg/dL)**					
Month 0	97.23, 98.27	125.57, 160.99	79.26, 40.06	86.85, 32.82	0.248
Month 3	99.39, 61.45	126.99, 84.85	77.96, 32.59	92.25, 44.56	**0.024**
Month 6	81.93, 34.84	87.25, 23.12	70.27, 32.81	87.98, 43.64	0.174
**HDL (mg/dL)**					
Month 0	60.44, 14.26	61.04, 10.50	60.66, 18.44	59.61, 13.33	0.944
Month 3	59.16, 13.31	58.01, 10.32	60.42, 16.68	59.10, 12.85	0.843
Month 6	57.70, 14.32	54.83, 12.21	58.84, 15.27	59.34, 15.45	0.539
**LDL (mg/dL)**					
Month 0	125.30, 26.37	129.80, 28.14	117.80, 25.27	128.29, 25.17	0.262
Month 3	126.58, 27.70	123.13, 27.06	124.40, 25.68	132.12, 30.49	0.517
Month 6	123.33, 32.56	121.39, 31.67	115.09, 35.58	133.05, 29.11	0.186
**LDL (Oxidized) (U/L)**					
Month 0	38.89, 11.30	41.32, 14.10	36.55, 10.69	38.82, 8.38	0.380
Month 3	44.28, 14.10	44.18, 13.56	45.90, 15.56	42.82, 13.67	0.778
Month 6	39.50, 14.46	36.71, 12.92	37.95, 16.52	43.64, 13.42	0.247
**ESR (mm/hr)**					
Month 0	22.64, 14.24	20.64, 10.89	21.45, 14.85	25.82, 16.50	0.438
Month 3	21.11, 13.96	17.77, 8.26	20.10, 15.80	25.41, 16.00	0.179
Month 6	20.41, 13.64	18.67, 10.53	18.05, 12.85	24.32, 16.46	0.253
**CD4 (cells/mm** ^**3**^ **)**					
Month 0	772.92, 236.59	790.95, 270.73	812.73, 171.79	715.09, 254.85	0.362
Month 3	808.77, 244.93	813.36, 242.67	836.71, 219.77	777.50, 275.89	0.732
Month 6	787.25, 232.78	751.90, 217.81	788.48, 219.61	819.82, 262.84	0.639
**CD8 (cells/mm** ^**3**^ **)**					
Month 0	552.20, 201.81	560.86, 158.28	588.05, 215.45	507.68, 226.04	0.412
Month 3	552.62, 202.54	552.77, 197.89	589.29, 216.07	517.45, 196.86	0.516
Month 6	546.94, 200.19	509.71, 169.50	565.14, 223.15	565.09, 208.28	0.590
**CD4/CD8**					
Month 0	1.69, 1.41	1.91, 2.32	1.54, 0.63	1.62, 0.50	0.659
Month 3	1.56, 0.53	1.57, 0.52	1.56, 0.64	1.56, 0.43	0.998
Month 6	1.56, 0.52	1.55, 0.64	1.51, 0.39	1.54, 0.51	0.938
**Quality of life**					
**Physical outcomes**					
Month 0	75.63, 14.30	75.72, 13.91	72.57, 15.57	78.60, 13.33	0.382
Month 3	80.69, 14.00	83.76, 13.74	81.54, 12.18	76.80, 15.50	0.246
Month 6	81.66, 13.19	85.46, 9.76	84.23, 11.50	75.58, 15.66	**0.025**
**Physical functioning**					
Month 0	77.12, 20.44	79.32, 16.35	68.64, 23.31	83.41, 18.99	**0.044**
Month 3	82.46, 15.89	88.64, 12.36	82.38, 15.78	76.36, 17.33	**0.035**
Month 6	86.17, 16.49	92.14, 9.43	87.14, 15.21	79.55, 20.70	**0.039**
**Physical role functioning**					
Month 0	88.07, 17.12	86.08, 19.95	86.08, 18.49	92.05, 11.92	0.417
Month 3	90.00, 16.75	90.34, 15.64	94.94, 9.40	84.94, 21.79	0.147
Month 6	91.21, 13.53	90.77, 12.12	95.54, 8.65	87.50, 17.47	0.148
**Body pain**					
Month 0	76.67, 21.06	75.00, 21.77	77.61, 20.81	77.39, 21.48	0.904
Month 3	81.04, 20.55	80.80, 22.89	79.76, 19.90	82.50, 19.53	0.910
Month 6	77.50, 21.38	82.14, 19.63	79.05, 18.90	71.59, 24.56	0.252
**General health perception**					
Month 0	60.67, 18.29	62.50, 21.17	57.95, 16.57	61.55, 17.34	0.692
Month 3	69.26, 17.64	75.27, 13.92	69.10, 16.02	63.41, 20.88	0.081
Month 6	71.75, 16.76	76.76, 15.77	75.19, 15.46	63.68, 16.53	**0.017**
**Mental outcomes**					
Month 0	71.99, 15.17	72.41, 16.66	69.98, 13.85	73.58, 15.35	0.730
Month 3	76.21, 13.15	77.20, 14.42	76.85, 11.81	74.63, 13.49	0.788
Month 6	77.80, 11.43	79.51, 12.36	80.42, 10.85	73.67, 10.35	0.108
**Vitality**					
Month 0	57.48, 16.92	58.24, 18.84	56.53, 14.62	57.67, 17.78	0.945
Month 3	64.04, 15.78	66.48, 15.36	65.48, 15.64	60.23, 16.31	0.377
Month 6	68.55, 15.31	71.13, 17.51	69.05, 15.99	65.63, 12.31	0.498
**Social role functioning**					
Month 0	77.65, 19.05	76.70, 22.59	76.70, 17.80	79.55, 17.05	0.854
Month 3	80.77, 17.41	84.66, 17.22	78.57, 19.02	78.98, 16.08	0.441
Month 6	81.05, 15.59	83.33, 13.88	84.52, 18.50	75.57, 13.07	0.121
**Role emotional**					
Month 0	86.62, 19.66	85.61, 19.28	83.71, 20.49	90.53, 19.47	0.501
Month 3	88.59, 17.53	83.33, 19.42	91.67, 14.91	90.91, 17.43	0.225
Month 6	88.93, 15.00	88.10, 15.94	95.24, 10.06	83.71, 16.36	**0.037**
**Mental health**					
Month 0	66.21, 16.24	69.09, 17.50	62.95, 14.20	66.59, 17.00	0.459
Month 3	71.46, 15.43	74.32, 16.71	71.67, 12.97	68.41, 16.36	0.452
Month 6	72.66, 14.42	75.48, 17.39	72.86, 11.68	69.77, 13.76	0.437

*Analysis of variance.

*Bold values indicate outcome values that were statistically significant, with a p-value < 0.05*.

### Primary Outcomes

Vitamin D and β-carotenoids levels increased in all three groups (Figure 2). Both VTL-7 and HCK observed a significantly higher increase in vitamin D than placebo, i.e., VTL-7 from 25.15 ± 2.13 to 35.53 ± 6.11 at month 3 (*p* < 0.001) and 33.38 ± 6.89 at month 6 (*p* < 0.001); HCK from 24.25 ± 3.08 to 28.43 ± 5.93 at month 3 (*p* = 0.005) and 27.40 ± 5.24 at month 6 (*p* = 0.012); and placebo from 24.00 ± 2.73 to 23.05 ± 4.39 at month 3 (*p* = 0.273) and 22.30 ± 6.23 at month 6 (*p* = 0.200). Similarly, β-carotenoids of VTL-7 vs. HCK groups significantly increased from 0.88 ± 0.68 vs. 0.94 ± 0.55 at baseline to 3.03 ± 1.79 (*p* < 0.001) vs. 1.09 ± 0.61 (*p* = 0.125) at month 3 and 3.26 ± 1.74 (*p* < 0.001) vs. 1.15 ± 0.66 (*p* = 0.064) at month 6, respectively. GEE analysis revealed a significantly higher increase in vitamin D (*p* < 0.001) and β-carotenoids (*p* < 0.001) in VTL-7 (capsule formulation) than HCK (granule formulation), both of which were significantly higher than the control groups (*p* < 0.001). Vitamin C, vitamin E, vitamin A, α-carotenoid, and coenzyme Q10 at months 3 and 6 did not increase significantly from baseline in any of the three groups.

### Secondary Outcomes

**Figure 3:**
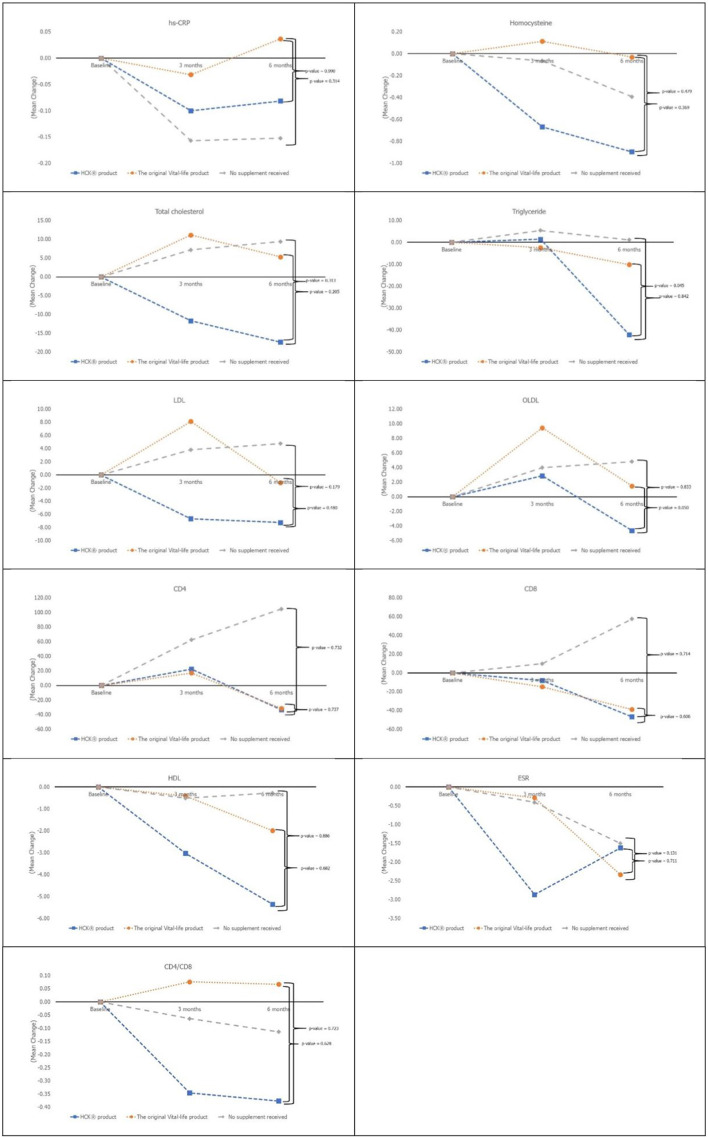
Secondary laboratory outcomes at baseline, month 3 and 6 among all 3 groups.

All of the secondary laboratory outcomes (hs-CRP, homocysteine, lipid profile, ESR, CD4, and CD8) and QOL did not increase significantly from baseline in any of the three groups (Figure 3). The overall QOL among the three groups in terms of physical health (*p* = 0.560) and mental health (*p* = 0.750) has increased but is not statistically significant.

## Discussion

Absorption of micronutrients could be affected by several factors such as variants of genetic polymorphisms, underlying health conditions, diet, exercise, smoking, alcohol consumption, age, and form of supplements (9). This quasi-experimental study revealed a comparably significant increase in serum levels of two out of the six micronutrients that were provided to the participants as supplements in two different oral formulations. The significant increase in only two out of the six micronutrients could be due to various reasons, including dose intake of these nutrients or individual absorption capabilities. Further and longer studies are needed to assess the reasoning behind a lack of significant increase among the other four micronutrients observed in this study.

The difference in laboratory results (vitamin D and beta carotene levels) between the study groups could be since vitamin D and beta carotenoid differed between the two products' formulations, representing two common types of Solid Oral Dosage Form (SODF). Some key differences among the differently formulated micronutrients include variations in the physicochemical state of the vitamin D (molecular forms, potency, and their physiological linkages), the complexity of the food matrix (the amount and type of fatty acids, dietary fibers, and presence/absence of vitamin D enhancer and inhibitor), and its interaction of other fat-soluble compounds with vitamin D, as well as the host-associated factors (e.g., age, disease, surgery, obesity, and genetic variation) (10). The homeostasis of vitamin C is influenced by several factors, including genetic polymorphisms and environmental and lifestyle factors, such as smoking and diet, as well as the presence of diseases (11). Excessive chronic alcohol intake is generally associated with vitamin deficiency (especially folate, thiamine, and vitamin B6) due to malnutrition, malabsorption, and ethanol toxicity. The effects of moderate alcohol use are mainly explained by a lower vitamin intake. In the case of vitamin A and beta-carotene, the effects on post-absorptive (lipoprotein) metabolism have been demonstrated (12).

Besides the effects of vitamins and minerals on blood biomarkers, previous studies showed that supplementation of multivitamin and mineral preparations has beneficial effects on mood and stress (13–15). In addition, the association between serum level of vitamin D and self-rated health in healthy male workers was observed (16). Moreover, results from a previous study revealed that elderly participants with poor physical health status assessed by the SF-36 exhibit lower alpha-tocopherol blood concentrations (17). The formulation type of the MVMM supplement used differed with each study but included both capsule and granule formulations. None of these studies, however, looked at the difference between MVMM formulation and its effect on the QOL. Nonetheless, our findings did not suggest that supplementation of vitamins and minerals may improve the QOL.

We observed that the participants who preferred no supplement were younger than the study groups. Previous studies revealed that supplement use increased with age, with 72% of adults of 65 years or older of age reporting an increased use compared with 40% of adults of 20–39 years of age (18). Several lifestyle and behavioral factors were associated with relatively less herb and dietary supplement use in young adults (19) for various reasons. First, they might prefer improving their diet and lifestyle rather than taking an oral supplement. Second, they might have been concerned about the potential side effects of the supplement (20).

The findings presented in this article could serve to influence the future of micronutrient supplementation, particularly for those with vitamin A or vitamin D deficiency. As the results from the GEE analysis (presented in the Results section) revealed a significantly higher increase in vitamin D and β-carotenoids in VTL-7 than HCK, consumers looking for supplementation of these nutrients may benefit more from capsule formulation than granule formulation. Knowing the composition of different MVMM formulations can help us to better understand the differences in the mechanism of action through which the nutrients are absorbed, based on the formulation type. Furthermore, expanding our knowledge of the different MVMM formulations can influence what kind of supplements are prescribed by medical providers to help with vitamin and mineral deficiencies, as well as help consumers to determine over-the-counter counter supplements to purchase. Still, more research should be done in this area to further explore the differences in efficacy between the two formulations by looking at micronutrients not included in this study.

This quasi-experimental study has some limitations. First and most important is the non-randomization nature of the study. Although a random allocation of participants is usually preferred, this study was conducted in a private international hospital setting, in which the participants were healthy and did not receive financial support from a third-party payer for this type of wellness service. Second, the different product formulations, which was the primary objective of this study, could indeed introduce another bias. That is, the participants who were familiar with conventional capsules, as opposed to the new formulation, were health-conscious. Although only two formulations/products were included, the findings from this study could be suggestive of the effect of supplement formulations on the change in laboratory parameters. Finally, it was not required that subjects follow a specific diet during participation in this study and, therefore, it cannot be ascertained that the diet of the subjects did not impact the uptake of CoQ10 or vitamins into the blood. Similarly, there was no method in place to assure that subjects took their supplementation exactly as prescribed, which could also influence the laboratory outcomes. Future studies should be conducted to control these factors.

## Conclusion

Micronutrient supplement formulation, specifically granule vs. capsule formulation, was found to impact certain laboratory outcomes but not QOL. More specifically, MVMM supplements in capsule formulation were found to increase the serum levels of some micronutrients, namely, vitamin D and β-carotenoids, to a higher extent than that of granule formulation. Nonetheless, participant adherence remains a potential confounder and should be further explored.

## Data Availability Statement

The original contributions presented in the study are included in the article/supplementary material, further inquiries can be directed to the corresponding author.

## Ethics Statement

The studies involving human participants were reviewed and approved by Bumrungrad International Institutional Review Board. The patients/participants provided their written informed consent to participate in this study.

## Author Contributions

AC conceived of and supervised the study. BT and PI collected and help to analyze the data. KP analyzed the data. PL and NT facilitate the data collection, data analysis, and supervised the study. KP, NJ, BT, PI, and JH drafted the manuscript. All authors read and approved the manuscript.

## Conflict of Interest

PI was employed by Asia Global Research Co., Ltd. The remaining authors declare that the research was conducted in the absence of any commercial or financial relationships that could be construed as a potential conflict of interest.

## Publisher's Note

All claims expressed in this article are solely those of the authors and do not necessarily represent those of their affiliated organizations, or those of the publisher, the editors and the reviewers. Any product that may be evaluated in this article, or claim that may be made by its manufacturer, is not guaranteed or endorsed by the publisher.
